# Unlocking high-performance HCl adsorption at elevated temperatures: the synthesis and characterization of robust Ca–Mg–Al mixed oxides

**DOI:** 10.1007/s11356-024-32752-w

**Published:** 2024-03-20

**Authors:** Jun Cao, Songshan Cao, Hualun Zhu

**Affiliations:** 1 National Engineering Research Center of Water Resources Efficient Utilization and Engineering Safety, Hohai University, Nanjing, 211111 China; 2 Center for Taihu Basin, Institute of Water Science and Technology, Hohai University, Nanjing, 211111 China; 3 Key Laboratory of Energy Thermal Conversion and Control of Ministry of Education, School of Energy and Environment, Southeast University, Nanjing, 210096 China; 4 Department of Chemical Engineering, University College London, London, WC1E 7JE UK

**Keywords:** Synthesis, Hydrotalcite-like compounds, HCl, SO_2_, Adsorption

## Abstract

The presence of HCl and SO_2_ gas imposes limitations on syngas utilization obtained from household waste in a wide range of applications. The hydrotalcite-like compounds (HTLs) have been proved that could remove HCl efficiency. However, the research on impact of synthesis conditions of HTLs and SO_2_ on HCl removal was limited. In this study, a range of Ca–Mg–Al mixed oxide sorbents was synthesized by calcining HTLs, with variations in crystallization temperature, solution pH, and the Ca/Mg molar ratio. These sorbents were examined for their effectiveness in removing HCl at medium–high temperatures under diverse conditions. The adsorption performance of selected sorbents for the removal of HCl, SO_2_, and HCl-SO_2_ mixed gas at temperature of 350 °C, 450 °C, and 550 °C, respectively, was evaluated using thermogravimetric analysis (TGA). It was observed that the HTL synthesis parameters significantly influenced the HCl adsorption capacity of Ca–Mg–Al mixed oxides. Notably, HTLs synthesized at 60 °C, a solution pH of 10–11, and a Ca/Mg ratio of 4 exhibited superior crystallinity and optimal adsorption characteristics. For individual HCl and SO_2_ removal, temperature had a minor effect on HCl adsorption but significantly impacted SO_2_ adsorption rates. At temperatures above 550 °C, SO_2_ removal efficiency substantially decreased. When exposed to a mixed gas, the Ca–Mg–Al mixed oxides could efficiently remove both HCl and SO_2_ at temperatures below 550 °C, with HCl dominating the adsorption process at higher temperatures. This dual-action capability is attributed to several mechanisms through which HTL sorbents interacted with HCl, including pore filling, ion exchange, and cation exchange. Initially, HCl absorbed onto specific sites created by water and CO_2_ removal due to the surface’s polarity. Subsequently, HCl reacted with CaCO_3_ and CaO formed during HTL decomposition.

**Graphical abstract:**

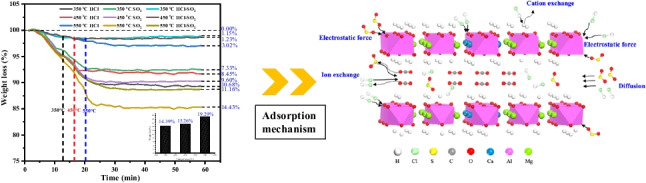

## Introduction

The challenge of achieving comprehensive treatment of household waste is becoming increasingly prevalent worldwide. Household waste has dual attributes of pollution and resources (Ye et al. 2020). With the promotion and implementation of the policy of waste classification, the calorific value of household waste has significantly increased (Nzihou et al. 2014). Consequently, there is a growing emphasis on the development of sustainable waste treatment methods that align with the objectives of carbon neutrality and achieving a carbon-zero environment (Lee et al. 2020; Díaz-Pérez & Serrano-Ruiz 2020). Pyrolysis and gasification have been recognized as promising methods for realizing the resource utilization of household waste (Grossman et al. 2019; Zhang et al. 2021). These technologies can not only reduce the weight and volume by decomposing, but also simultaneously contribute to energy conservation and acquisition of valuable products: syngas, bio-oil, and biochar (Aswathi et al. 2022; Wang et al. 2023; Xie et al. 2022).

The syngas obtained can serve various purposes. It can act as a substitute for traditional natural gas and coal in power generation and heating, thereby reducing dependence on fossil fuels (Jakobs et al. 2013). A portion of the syngas can be converted to hydrogen through steam reforming reactions, which can be used in fuel cells and other hydrogen-based energy technologies (Mann 1995). Additionally, syngas can also serve as a raw material for chemical production, including the synthesis of methanol, ammonia, ethylene, and other chemicals, which are widely used in the chemical industry (Alnouss et al. 2021; Singh et al. 2022; Zheng et al. 2022). However, during pyrolysis/gasification, chlorine-containing products like plastic, fabric, and rubber products are predominantly converted to HCl with concentrations ranging from several to hundreds even thousand mg/m^3^ (Pan et al. 2021; Truc and Lee 2019) in syngas. The high concentration of HCl in syngas can impose limitations on the key parameters of power generation, resulting in low efficiency (Lee et al. 2013; Wang et al. 2020). It can also lead to surface and pipe corrosion due to high temperature and low temperature, as well as adversely affect the performance of molten carbonate (MCFC) and solid oxide (SOFC) fuel cells (Błesznowski et al. 2013; Krishnan et al. 1999). Besides industrial issues, HCl is also the third-largest source of global anthropogenic acidification, following SOx and NOx emissions (Murciano et al. 2011; Tan et al. 2020), which not only poses threats to the environment but also has negative impacts on human health (Bjoerkman and Stroemberg 1997; Li et al. 2019). Consequently, there is a compelling necessity to remove HCl and enable efficient syngas utilization in a wide range of applications. The regulations suggest that the gas concentrations of HCl should be maintained below 16.3 mg/m^3^ in flue gas (Kuramochi et al. 2005).

Various low-cost adsorbents, including Ca-based (Li et al. 2015b; Liu et al. 2022; Liu 2005; Mizukoshi et al. 2007), Na-based (Lee et al. 2003; Liang et al. 2018; Verdone and De Filippis 2006), and others (Liu et al. 2021), have been studied for HCl removal, typically at reaction temperature below 150 °C. However, the temperatures of syngas often exceed 400 °C, reaching up to 1000 °C, which means there would be a loss in efficiency when cooling syngas from medium-high temperature to 150 °C or even room temperature. Additionally, at 250 to 450 °C, the high concentration of HCl can enhance the toxicity of PCDD/Fs (Li et al. 2015a; Lundin et al. 2013). Many such sorbent materials exhibit unsatisfactory adsorption capacity, especially at high temperatures with the HCl adsorption capacities of these materials decreasing drastically as temperature rises (Cao et al. 2018a; Chibante et al. 2010; Shemwell et al. 2001). Consequently, recent research has focused on developing efficient sorbents with high adsorption capacity at medium-high temperatures, especially those exceeding 450 °C. In recent years, many studies have been devoted to investigating the catalytic abilities of hydrotalcite-like (HTLs) in various applications, including catalysis, catalyst precursors or catalyst supports, anion exchangers, and their utilization in fields such as medicine and biochemistry (Cota et al. 2010a, b; Hoyo 2007). Hydrotalcite-type materials are considered as promising candidates because of their high adsorption capacity for anionic species (Hamouda et al. 2018). Kameda et al. found that Mg–Al layered double hydroxide could react with hydrochloric acid and HCl in gas at 140 ~ 200 °C (Kameda et al. 2020). Our previous study has also demonstrated the potential of HTL derivatives, such as Mg–Al HTLs, Ca–Mg–Al HTLs, Zn–Mg–Al HTLs, and Mg–Fe HTLs, which were self-prepared, used to remove HCl with high adsorption capacity at medium-high temperature (350 ~ 700 °C) (Cao et al. 2018a, b, 2014, 2024). However, previous studies have primarily focused on structural changes, metal ion variations, and the influence of different operating conditions on their adsorption performance, with limited attention given to the impact of synthesis conditions of HTLs. In the other researchers’ work (Elwakeel 2010; Elwakeel et al. 2020a, b), it is emphasized that the conditions under which synthesis occurs significantly influence the performance of adsorbents. Furthermore, the interaction between SO_2_ and HCl removal using the Ca–Mg–Al mixed oxide sorbent, synthesized via hydrotalcite-like compounds (HTLs), has been explored to a relatively limited extent.

Therefore, to maximize syngas resource utilization and develop a highly efficient HCl adsorbent, this study investigates the effects of synthesis and characterization of Ca–Mg–Al mixed oxide sorbents on HCl removal from syngas. The study is centered around two primary objectives. The first objective aims to optimize the sorbent by varying synthesis conditions, including crystallization temperature, solution pH value, and the Ca/Mg molar ratio, to assess their impact on adsorption capacity. The second objective focuses on assessing the influence of SO_2_ on HCl removal using the premier sorbent, which was selected through self-conducted thermogravimetric analyses.

## Material and experimental method

### Materials and synthesis of HTLs

The raw materials used to prepare the sorbent in this study were all analytical-grade reagents. Mg (NO_3_)_2_·6H_2_O, Ca (NO_3_)_2_·4H_2_O, and Al (NO_3_)_3_·9H_2_O were purchased from Longxi Chemical Reagent Factory in China. NaHCO_3_ and Na_2_CO_3_ were purchased from Shanghai Lingfeng Chemical Reagent Factory in China. HCl (1500 mg/m^3^), SO_2_ (2000 mg/m^3^), and N_2_ (99.999%) were obtained from Nanjing Shangyuan Gas Product Co. Ltd., China.

**Fig. 1:**
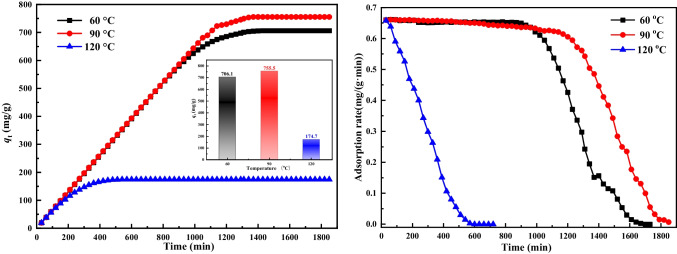
Adsorption capacity and adsorption rate of HTLs prepared under different crystallization temperatures: Reaction temperature of 550 °C, N_2_ atmosphere, initial HCl concentration of 750 mg/m^3^, flow rate of 0.9 L/min, and sorbent mass of 1 g

In the chemical formula for HTLs, M^2+^ always represents Mg^2+^, Fe^2+^, Co^2+^, Ca^2+^, Sr^2+^, Ba^2+^,…; M^3+^ represents Al^3+^, Cr^3+^, Fe^3+^,…; A^*n*−^  = CO_3_^2−^, Cl^−^, SO_4_^2−^,…, and *x* varies between 0.2 and 0.33 (Cavani et al. 1991). In this study, Ca–Mg–Al HTLs were synthesized through a co-precipitation method, using Ca^2+^ to partially substitute Mg^2+^ (Cao et al. 2014). Calcium was used as an active ingredient to remove HCl. A determined amount of Mg (NO_3_)_2_·6H_2_O, Ca (NO_3_)_2_·4H_2_O, and Al (NO_3_)_3_·9H_2_O were dissolved in a solution at a certain temperature. A solution containing NaHCO_3_ and Na_2_CO_3_ was prepared as a precipitant. The two solutions were titrated into the same vessel in 3 min simultaneously, while maintaining an alkaline solution. The resulting mixture was stirred continuously and aged for 30 min. After that, the sediment was isolated by filtering the resulting suspension, followed by multiple washes with deionized water until the value of solution pH reached 7. The samples obtained were dried for 24 h and calcined at 350 °C for 2 h. The grain size was 0.45 mm. The detailed synthesis process is shown in Fig. 1, and synthesis conditions are listed in Table 1.

**Table 1:** HTLs synthesis conditions

Parameter	Unit	Value
Crystallization Temperature	°C	60, 90, and 120
pH of solution	\	9 ~ 10, 10 ~ 11
Molar ratio of Ca/Mg	\	1/3, 1/2, 1/1, 2/1, 3/1, 4/1
Calcined Temperature	°C	350
Drying time	h	24

### Experimental and test processing

#### Adsorbent performance testing experiment

The HCl removal efficiencies of samples were tested in an adsorbent performance system (Cao et al. 2018b). This system comprises three major sections: gas application, performance test, and HCl analysis section. The gas application section consists of N_2_ and HCl. Each gas stream is controlled by a mass flow meter. A portion of N_2_ is used to mix with HCl to obtain the desired initial HCl concentration, while the remaining N_2_ serves as balance gas. The performance test section is equipped with a fixed bed reactor and electrical heating system, and the accuracy of the temperature controller is ± 1 °C. HCl concentration measurements are conducted by an online HCl analyzer (Model 7900FM, GFC, Signal Co., Britain), which is employed after proper filtration and drying. The accuracy in calibration of the gas analyzer is 0.03 mg/m^3^.

The experiments to determine the adsorption capacities of calcined HTLs samples were carried out as follows. An amount of sample (1 g) was placed at the center of the quartz glass tube within a fixed bed reactor. N_2_ was pumped in with a flow rate of 110 ml/min for 30 min after thorough leak checking. Subsequently, the reactor was gradually heated from room temperature to 550 °C. The desired concentration of HCl was obtained by mixing pure gases (HCl and N_2_). The residual HCl gas concentration was continuously monitored and analyzed after condensation and filtration. Gases were safely released into the environment through the NaOH adsorption solutions.

Each test was performed in identical conditions to guarantee the accuracy of the experimental results, and the margin of error between replicated experiments was carefully controlled and maintained at a level below 3%.

#### Thermogravimetric analysis

Thermogravimetric analyses were conducted using a self-built pressurized thermogravimetric system, designed to mitigate HCl corrosion at various temperatures and atmospheres. Within the system, the sorbent was placed in the middle of the reactor, equipped with a sensitive weighing mechanism that had a sensitivity of 8 mV/μm, based on eddy current dampening. The TGA analysis was first performed under a flow of N_2_ from room temperature to 350 °C, 450 °C, and 550 °C, respectively. Upon reaching the designated temperature, the gas environment was switched to one of the reaction gases, including HCl (1500 mg/m^3^), SO_2_ (2000 mg/m^3^), and HCl-SO_2_ mixed gas (HCl: 1500 mg/m^3^, SO_2_: 2000 mg/m^3^), respectively. These obtained samples were denoted as *T*-*i*, where *T* represented the reaction temperature in °C, *i* indicated the specific gas used, HCl, SO_2_, HCl–SO_2_, respectively.

#### Experimental data processing

The adsorption capacity of the samples ${q}_{t}$ was calculated using Eq. (1):

1 $${q}_{t}=mgHCl/gHTLs={10}^{-3}\underset{0}{\overset{t}{\int }}\frac{({C}_{0}-{C}_{t})V}{{m}_{HTLs}}dt$$

where *V* is the flow rate of flue gas, L/min; ${C}_{0}$ is initial HCl concentration, mg/m^3^; ${C}_{t}$ is the outlet concentration of HCl at time *t*, mg/m^3^; ${m}_{HTLs}$ is the mass of HTLs, g; $t$ is reaction time, min. The ${q}_{t}$ is the total amount of adsorption of HCl adsorbed on the HTLs from the beginning to *t* moment.

The adsorption rate $k$ is the parameter that reflects the degree of reaction in the reaction of removal of HCl and was obtained by Eq. 2:

2 $$k=\frac{\Delta {C}_{t}}{\Delta t}$$

where $k$ is adsorption rate, mg/(g min); $\Delta t$ is the gas and sorbent contact time, min; $\Delta {C}_{t}$ is quality of the reacted HCl, mg/g. And $\Delta {C}_{t}$ was given by Eq. 3:

3 $$\Delta {C}_{t}=\underset{0}{\overset{t}{\int }}\frac{({C}_{0}-{C}_{t})\cdot {10}^{-3}\cdot V}{{m}_{HTLs}}dt$$

Substitute Eq. 3 to Eq. 2, and the $k$ was estimated from the follow relations:

4 $$k=\frac{\underset{0}{\overset{t}{\int }}\frac{({C}_{0}-{C}_{t})\cdot {10}^{-3}\cdot V}{{m}_{HTLs}}dt}{\Delta t}$$

### Characterization methods

Prior to HCl adsorption, a comprehensive physicochemical characterization of selected HTLs was undertaken. To study the structural patterns of the HTLs, X-ray powder diffraction (XRD) over 2θ ranges from 5 to 80° was performed using a D8 Advance Diffractometer (Bruker Axs Ltd. Germany) with Cu-Kα radiation at 40 kV and 100 mA. The diffraction patterns of all samples were conducted at room temperature under a nitrogen atmosphere. The alterations in functional groups within the most effective HTLs, before and after HCl adsorption, were recorded from 4000 to 400 cm^−1^ using Fourier transform infrared spectroscopy (FTIR, Brucker Model Vextor 22, Germany). The powdered samples were mixed with KBr and subsequently pressed in the form of pellets for the measurement.

## Results and discussion

### The effect of synthesis conditions of HTLs

To achieve the optimal adsorption performance of HTLs, this study systematically examined the impact of structural factors on HCl sorption. The HTL materials were prepared by varying the crystallization temperatures, the values of pH, and the molar ratios of Ca/Mg.

#### The effect of crystallization temperature

The product crystal pattern always plays a decisive role in defining its inherent characteristics. Improved crystalline forms often result in enhanced product qualities. The crystallization temperature and crystallization time significantly contribute to the formation of crystalline, with crystallization temperature having a more pronounced impact. Consequently, this study mainly discusses the effect of crystallization temperature on the properties of the samples.

The effects of crystallization temperature of 60, 90, and 120 °C, respectively, on the adsorption capacity and adsorption rates were investigated under specific conditions: a reaction temperature of 550 °C, a N_2_ atmosphere, an initial HCl concentration of 750 mg/m^3^, a flow rate of 0.9 L/min, and a sorbent mass of 1 g. The results are shown in Fig. 1. During the initial 1000 min, the adsorption capacities and adsorption rates of HTLs obtained at 60 and 90 °C (HTLs-60 and HTLs-90) exhibited similar values. Specifically, HTLs-60 displayed capacities of 663.49 mg/g and an adsorption rate of 0.61 mg/(g min), while HTLs-90 exhibited capacities of 662.23 mg/g and an adsorption rate of 0.63 mg/(g min). Subsequently, the adsorption capacity of HTLs-60 tended to saturation, the adsorption rate followed a similar trend, reaching a final capacity of 706.07 mg/g at 1500 min. In contrast, the adsorption capacity of HTLs-90 continued to increase and reached up to 755.49 mg/g at 1820 min. The decrease in capacity rate observed at 1200 min indicated that, initially, both HTLs obtained at 60 and 90 °C demonstrated promising characteristics in terms of the removal of HCl, with HTLs-90 showing superior performance. However, the adsorption capacity and adsorption rate of the sample synthesized at crystallization temperature of 120 °C (HTLs-120) consistently exhibited lower adsorption capacity and rate compared to HTLs-60 and HTLs-90 throughout the experiments. Interestingly, when the crystallization temperature was 90 °C, the formation of metal oxides and mental nitrogen oxides occurred. These impurities might enhance the adsorption of HCl by HTLs, albeit potentially compromising the mechanical integrity of the sorbent. This phenomenon could be attributed to the relatively slower formation rate of crystalline grains at lower crystallization temperatures, resulting from reduced ion movement rates, leading to smaller crystal sizes, lower crystallinity, and the formation of impure phases. Conversely, elevating the crystallization temperature facilitated the accelerated formation of crystalline grains, culminating in more comprehensive crystalline structures and a reduction in impurities. Nevertheless, a further increase in temperature beyond 90 °C led to an excessively rapid rise in the kinetic energy of the hydrotalcite molecules, which proved detrimental to the formation of stable particles. As a result, a crystallization temperature of 60 °C was identified as the optimal condition for achieving the desired structural integrity and adsorption efficiency.

#### The effect of pH of the solution

Given that Al^3+^ readily precipitates as hydroxide within a pH range of 3.9 to 8, the preparation process of HTL materials is particularly prone to the formation of various impure phases if the solution’s pH falls below 9. Therefore, to mitigate this risk and ensure purity, the HTLs were synthesized within pH ranges of 9 to 10 and 10 to 11.

**Fig. 2:**
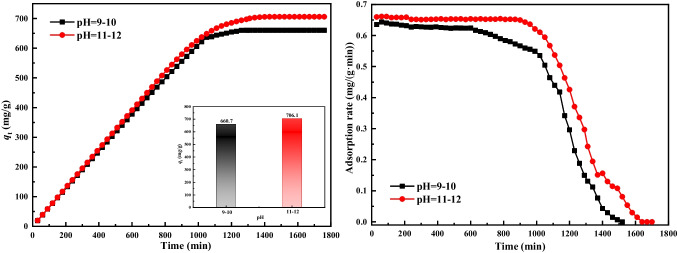
Adsorption capacity and adsorption rate of HTLs prepared under different pH values: Reaction temperature of 550 °C, mixed gas flow rate of 0.9 L/min, initial HCl concentration of 750 mg/m^3^, and sorbent mass of 1 g

**Fig. 3:**
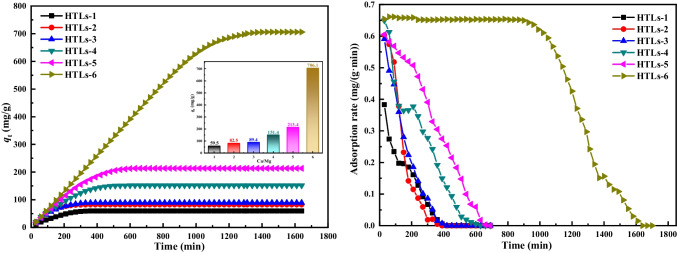
Adsorption capacity and adsorption rate of HTLs prepared under different molar ratios of Ca/Mg: at 550 °C, mixed gas flow rate of 0.9L/min, initial HCl concentration of 750 mg/m^3^, and sorbent mass of 1 g

Figure 2 illustrates the adsorption capacities and adsorption rates of HTLs obtained at pH levels of 9 ~ 10 and 10 ~ 11 (referred to HTLs-p10 and HTLs-p11, respectively). These experiments were conducted under standardized conditions, including a reaction temperature of 550 °C, a mixed gas flow rate of 0.9 L/min, an initial HCl concentration of 750 mg/m^3^, and a sorbent mass of 1 g. As shown in Fig. 3, a rise in the pH level during the synthesis process resulted in HTLs exhibiting enhanced adsorption capacities. The highest adsorption capacity of HTLs-p11 was up to 706.07 mg/g, with an adsorption rate of 0.66 mg/(g min). These values significantly surpassed those of HTLs-p10, which had an adsorption capacity of 660.67 mg/g and an adsorption rate of 0.64 mg/(g min). This outcome can be ascribed to the observation that maintaining the pH value within the range of 10 to 11 yields HTLs with superior structural integrity, notably characterized by an increased specific surface area. Upon calcination at high temperatures, these HTLs transform into mixed metal oxides, exhibiting significantly enlarged surface areas, which in turn augment the adsorption capacity of the materials. Consequently, a pH range of 10 to 11 has been determined as the optimal condition for synthesizing HTLs, balancing structural quality with functional performance.

#### Effect of molar ratio of Ca/Mg

Figure 3 presents the adsorptions capacities and adsorption rates of HTLs with varying Ca/Mg ratios, including 1/3, 1/2, 1/1, 2/1, 3/1, and 4/1, designated as HTLs-1 through HTLs-6, respectively, employing a mixed gas flow rate of 0.9 L/min, an initial HCl concentration of 750 mg/m^3^, and a sorbent mass of 1 g. It reveals that the adsorption capacities of hydrotalcite-like (HTL) materials varied significantly, ranging from 59.48 to 706.07 mg/g, as the Ca/Mg ratio was adjusted from 1/3 to 4/1. A parallel trend was observed in the adsorption rates of the HTL materials. Specifically, when the Ca/Mg ratio was below 4, the initial adsorption rates were relatively low and dropped rapidly. Notably, the initial adsorption rate was only 0.4 mg/(g min) at a Ca/Mg ratio of 1/3. Conversely, HTLs-6 demonstrated the highest initial adsorption rate, approximately 0.66 mg/(g min), which was sustained for approximately 900 min. These findings suggest that incorporating Ca enhances the adsorption capabilities of HTLs to a certain extent, yet an excessive concentration of Ca may detrimentally affect the dechlorination process by altering the layered structure of the sorbents. Therefore, these results highlight that the efficiency of sorbent adsorption is determined not only by the underlying chemical reactions but is also significantly influenced by the compositional makeup of the materials.

### The characteristic of HTLs

**Fig. 4:**
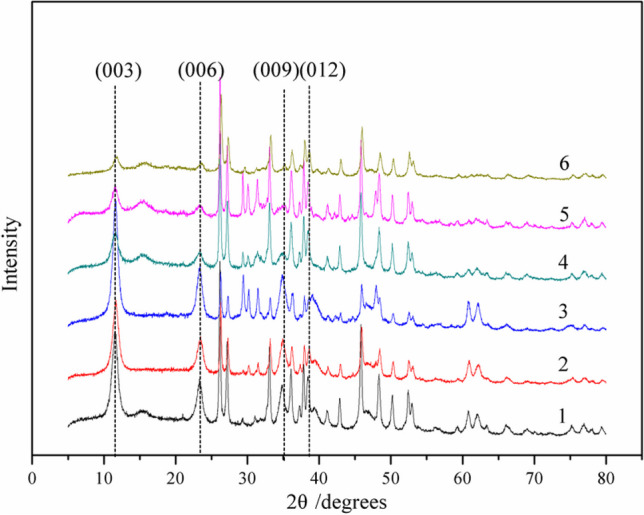
Power XRD patterns of HTLs samples

The structural characteristics of the HTLs (denoted as pristine HTLs-1 through HTLs-6) were elucidated through X-ray diffraction (XRD) patterns, as depicted in Fig. 4. The characteristic spacing of the (003) planes for HTLs-1 ~ 6 is tabulated in Table 2. Figure 4 distinctly showcases characteristic diffraction peaks indicative of a hydrotalcite-like structure, with well-defined Bragg reflections from the typical planes of (003), (006), (009), and (012), thereby confirming the formation of HTL phases in the synthesized samples. The sharp and pronounced nature of these peaks signifies the exceptional crystallinity of all samples. Notably, an increase in calcium content was observed to reduce the intensities of these characteristic peaks, a phenomenon that can be ascribed to the altered interactions between various laminar cations and the interlayer anions. The ionic radius of Ca is larger than that of Mg, resulting in weaker interactions between calcium and the interlayer anions. Consequently, excessive calcium content can disrupt the layered structure. However, it is important to note that the adsorption capacities of HTLs did not exhibit straightforward alterations that corresponded directly to changes in their structures. Instead, it has been observed that maintaining a constant molar ratio of (Ca + Mg)/Al unveils an optimal Ca/Mg molar ratio that enables HTLs to achieve heightened HCl adsorption capacities.

**Table 2:** d003 and suggested formula of different molar ratios of Ca/Mg

Sample	Ca/Mg	Crystallization Temperature	pH	*d*_003_/(Å)	Suggested formula
1	1/3	60 °C	10–11	7.62	Ca_0.19_Mg_0.57_Al_0.24_(OH)_2_(CO_3_)_0.12_·0.765H_2_O
2	1/2	60 °C	10–11	7.60	Ca_0.25_Mg_0.51_Al_0.24_(OH)_2_(CO_3_)_0.12_·0.765H_2_O
3	1/1	60 °C	10–11	7.57	Ca_0.38_Mg_0.38_Al_0.24_(OH)_2_(CO_3_)_0.12_·0.765H_2_O
4	2/1	60 °C	10–11	7.51	Ca_0.51_Mg_0.25_Al_0.24_(OH)_2_(CO_3_)_0.12_·0.765H_2_O
5	3/1	60 °C	10–11	7.49	Ca_0.57_Mg_0.19_Al_0.24_(OH)_2_(CO_3_)_0.12_·0.765H_2_O
6	4/1	60 °C	10–11	7.47	Ca_0.608_Mg_0.152_Al_0.24_(OH)_2_(CO_3_)_0.12_·0.765H_2_O

Table 2 lists the characteristic spacing of the (003) plane, the thickness of the unit layer corresponding the spacing of the (003) plane (denoted as *d*_003_) (Toraishi et al. 2002), in addition to the proposed formulas for HTLs-1 through HTLs-6. As delineated in Table 2, the minimal *d*_003_ value among the samples, measuring 7.47 Å, was recorded for HTLs-5. According to the adsorption capacity in Fig. 3, HTLs-5 demonstrated the lowest HCl adsorption capacity among all samples despite possessing a Ca/Mg molar ratio of 3/1. This suggested that the structural characteristics of HTLs sorbents might play a crucial role in removing HCl. In contrast, HTLs-6, with the largest *d*_003_ spacing, corresponded to the most elevated HCl adsorption capacity, underscoring a direct correlation between increased interlayer spacing and adsorption performance. The *d*_003_ values for HTLs-1 ~ 4 exhibited minimal variation, recorded at 7.62, 7.60, 7.57, and 7.51 Å, respectively, highlighting the nuanced relationship between structural parameters and functional outcomes. These samples exhibited similar trends in their adsorption capacities, indicating the importance of pore filling in HCl removal. Although HTLs-2 and HTLs-4 had identical *d*_003_ value, HTLs-4 demonstrated superior adsorption capacities compared to HTLs-2. This discrepancy could be attributed to the combined influence of both the Ca^2+^ content and the structural characteristics of the sorbent, which enhance both the pore filling and ion exchange. Above all, it is evident that the sorbent’s HCl adsorption capacity is influenced by both the Ca/Mg molar ratio and the sorbent’s structural features. Notably, a Ca/Mg molar ratio of 4 emerges as the most conducive for achieving the optimal HCl adsorption capacity.

**Fig. 5:**
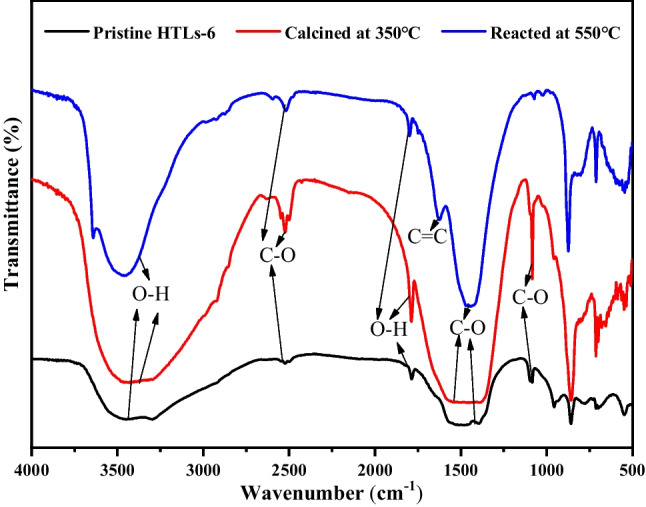
FTIR spectra of HTLs-6 before and after HCl adsorption

To discern alterations in the functional groups of the samples, FTIR spectroscopy was employed. As shown in Fig. 5, the broad peak observed in the range of 3000 to 3750 cm^−1^ was attributed to the stretching vibrations of O–H groups. These groups are associated with brucite-like layers and interlayer water molecules, confirming their presence in the analyzed samples (Chebout et al. 2010; Wang et al. 2010). Additionally, the bending vibration of the interlayer water was also reflected in the range of 1640 ~ 1750 cm^−1^. The carbonates exhibited characteristic bands associated with various modes of infrared-sensitive vibrations of the anion. The absorption bands at 2500 cm^−1^ implied the presence of free CO_3_^2−^ anions. Compared with the pristine HTLs-6, the intensity of the free CO_3_^2−^ peak in calcined HTLs-6 at 350 °C was stronger. However, when the calcined sample reacted with HCl at 550 °C, the intensity of peak diminished. This phenomenon could be attributed to the partial decomposition of HTLs-6 during calcination, resulting in the production of CaCO_3_ and an increased presence of free CO_3_^2−^, which enhanced the cation exchange with Cl^−^. Following the reaction with HCl, CaCO_3_ was consumed, leading to the changes of vibrations in the range of 1100 ~ 1200 cm^−1^. The intercalated CO_3_^2−^ anions contributed to the intense adsorption band at 1340 ~ 1550 cm^−1^. The narrowing of the peak was attributed to the partial decomposition of HTLs and the reaction with HCl, which diffused into the interior of the sorbent. Especially, C = C band at 1636 cm^−1^ was observed in the sorbent at 550 °C, indicating the rearrangement of carbonate anions. This observation is attributed to the presence of both CaCO_3_ and CaO in the samples obtained through the calcination of HTLs. The initial diffusion of HCl into HTLs, followed by reactions with CaCO_3_ and CaO, revealed that HCl adsorption on HTLs was affected by diffusion, cation exchange, ion exchange, and electrostatic force. Furthermore, the presence of competing impurity anions in the system may vie with HCl for adsorption sites on the HTL sorbent. Ultimately, the analyzed samples demonstrated distinct alterations under varying temperature regimes as evidenced by the findings and corroborated by our preceding research (Cao et al. 2018a), detailed as follows (Reactions 5–7):

5 $$\begin{array}{c}{\text{Ca}}_{0.608}{\text{Mg}}_{0.152}{\text{Al}}_{0.24}{\left({\text{OH}}\right)}_{2}{\left({\text{CO}}_{3}\right)}_{0.12}\cdot 0.765{\text{H}}_{2}{\text{O}}\stackrel{350^\circ {\text{C}}}{\to } \, \text{Ca-Mg-Al HTLs}\\ +{\text{Ca-Mg-Al mixed oxides+CaCO}}_{3}+{\text{CO}}_{2}+0.765{\text{H}}_{2}{\text{O}}\end{array}$$

6 $$\text{Ca-Mg-Al HTLs}\stackrel{450-550^\circ {\text{C}}}{\to }{\text{CaCO}}_{3}+{\text{CaO+CO}}_{2}{+ \text{H} }_{2}{\text{O}}+\text{Ca-HTO}$$

7 $${\text{CaCO}}_{3}\stackrel{\ge 550^\circ {\text{C}}}{\to }{\text{CaO+CO}}_{2}$$

Furthermore, when reacting with HCl at 550 °C, in addition to the diffusion and electrostatic force, the gas mainly reacts with HTO through the following chemical reactions (Reactions 8–11):

8 $$\text{Ca-Mg-Al }\text{HTLs+HCl}\to {\text{Ca-Cl HTLs+CO}}_{2}{+ \text{H} }_{2}{\text{O}}$$

9 $$\text{Ca-Mg-Al mixed }\text{oxides+HCl}\to {\text{Ca-Mg-Al mixed chloride+H}}_{2}{\text{O}}$$

10 $${\text{2CaCO}}_{3}+ \text{3HCl} \to {\text{CaCl}}_{2}{+ \text{H} }_{2}{\text{O+CaClOH+2CO}}_{2}$$

11 $$\text{CaO+2HCl}\to {\text{CaCl}}_{2}{+ \text{H} }_{2}{\text{O}}$$

In summary, the optimal conditions for synthesizing HTLs with the highest HCl removal capacity involve an aging temperature of 60 °C, a pH value of 10–11, and a mole ratio of Ca/Mg of 4/1.

Comparative analyses between previous investigations on HCl removal and the findings of this work are delineated in Table 3. For the augmentation of the adsorbent’s cost-effectiveness, it is imperative to consider both the adsorption capacity and the duration of sustainable adsorption. The data presented in the table elucidates that hydrotalcite and hydrotalcite-like sorbents manifest comparatively elevated dechlorination efficacy. Notably, the Ca HTLs, upon achieving optimal synthesis conditions, delineate the paramount adsorption characteristics for HCl, with a capacity of 706.07 mg/g and a prolonged efficacy duration of 1000 min.

**Table 3:** Comparison of dechlorination performance of different sorbents in the literature

Sorbent	Reaction temperature (℃)	Adsorption capacity (mg/g)	Adsorption efficiency (%)	Time (min)	Literature
NaHCO_3_	550	−	99	74	Cao et al. (2014)
MgO	550	−	82.6	10	Cao et al. (2014)
CaO	550	−	85.5	200	Cao et al. (2018a)
Hydrotalcite	170		93	90	Kameda et al. (2020)
Hydrotalcite	300	120.1	90.11	120	Cao et al. (2024)
Fe Hydrotalcite-like	550	−	96.96	550	Cao et al. (2018b)
Zn Hydrotalcite-like	550	−	96.9	456	Cao et al. (2018a)
Ca Hydrotalcite-like	550	706.07	−	1000	This work

### Thermogravimetric experiments

**Fig. 6:**
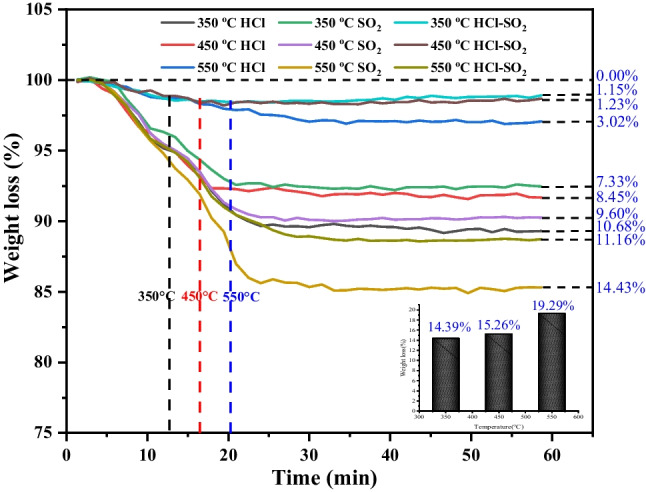
Adsorption of HCl on selected HTLs as a function of gas atmosphere

Figure 6 illustrates thermogravimetric analysis of selected sorbents during the removal of HCl, SO_2_, and a mixed gas of HCl-SO_2_ at temperature of 350 °C, 450 °C, and 550 °C, respectively. In experiments conducted solely in N_2_ atmosphere, the weight loss observed in the Ca–Mg–Al HTL samples exhibited a temperature-dependent decrease: 14.39% at 350 °C, transitioning to 15.26% at 450 °C, and further to 19.29% at 550 °C. The observed decline in weight loss with increasing temperature suggests a diminution in the reactivity of the samples. Conversely, the introduction of reaction gases—HCl, SO_2_, and a mixture of HCl-SO_2_—resulted in a decrease in weight loss for all Ca–Mg–Al HTL samples under identical conditions, indicative of chemical reactions occurring between the samples and the gases. Notably, the weight loss curves for reactions involving HCl and SO_2_ exhibited divergent trends, reflecting distinct interactions with the Ca–Mg–Al HTLs. As the temperature increased from 350 to 550 °C, a corresponding decrease in the weight loss of the sorbent was observed, from 10.68% at 350 °C to 3.02% at 550 °C, indicating that the sorbent’s adsorption capacity for HCl enhances with rising temperature. Such an observation aligns with the outcomes of prior research in this field (Cao et al. 2018a). Conversely, as the temperature was elevated from 350 to 550 °C, the adsorption capacity of the sorbent for SO_2_ diminished, evidenced by an increase in the weight loss rate from 7.33% at 350 °C to 14.43% at 550 °C. At the lower temperature of 350 °C, the sorbent demonstrated higher removal efficiency for SO_2_ compared to HCl. However, with a rise in temperature to 450 °C, the removal efficiency for HCl not only remained high but also exceeded that of SO_2_. Notably, at 550 °C, there was a significant decline in the removal efficiency for SO_2_. These findings indicated that the reaction temperature exerted a limited influence on the removal of HCl by Ca–Mg–Al HTLs, in contrast to its effect on SO_2_ removal (Cao et al. 2014). When both HCl and SO_2_ were simultaneously introduced to the Ca–Mg–Al HTLs, the weight loss of sample slightly increased as the temperature rose from 350 to 450 °C, going from 1.15 to 1.23%. However, as the temperature continued to increase, the weight loss of sample rapidly escalated to 11.16%. This suggested that SO_2_ had a minimal effect on the simultaneous adsorption of HCl and SO_2_ by Ca–Mg–Al HTLs at temperatures below 550 °C. The Ca–Mg–Al HTLs demonstrated the ability to remove both HCl and SO_2_ simultaneously with high removal efficiency. This capacity was particularly pronounced at elevated temperatures, reaching a peak at 550 °C, as underscored by a marked increase in sample weight loss indicative of intensified adsorption. The results also showed that the presence of HCl gas at 550 °C notably enhanced the adsorption efficiency for SO_2_, suggesting a synergistic interaction between the removal processes of these gases at high temperatures.

**Fig. 7:**
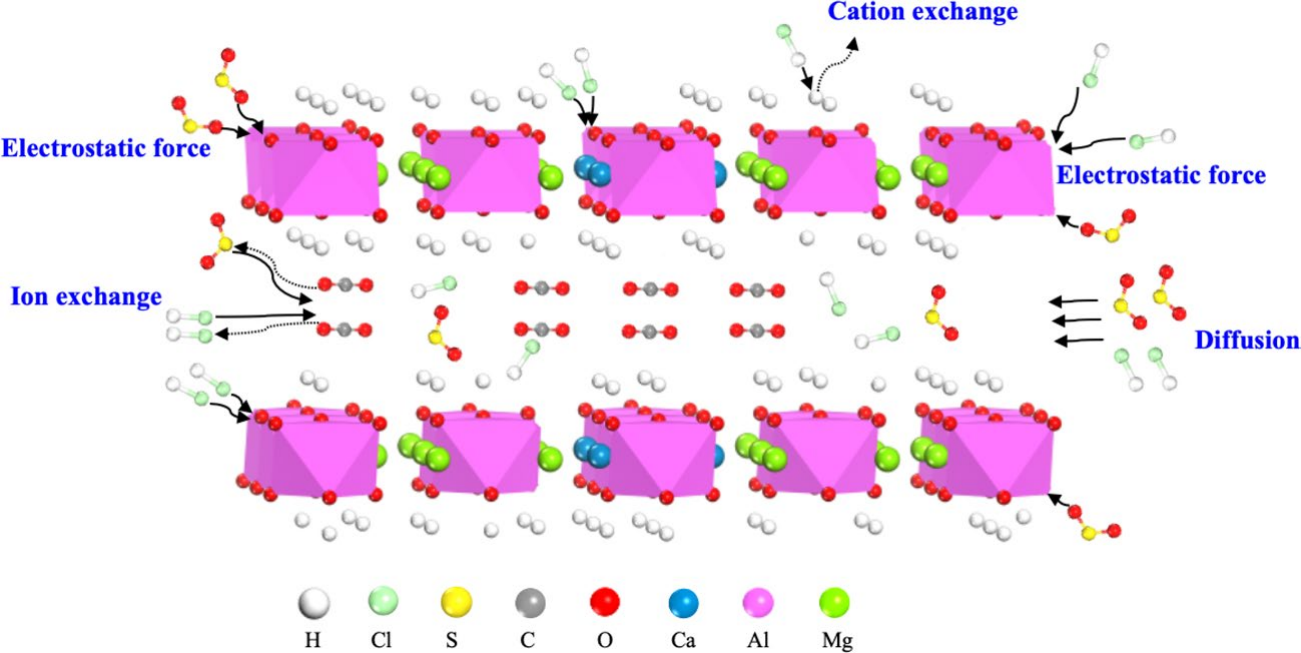
The adsorption mechanisms of sorbent for HCl and SO_2_

The adsorption mechanisms by which the sorbent captures acid gases are depicted in Figure 7. At temperatures of 350 °C and 450 °C, the sample primarily consisted of Ca–Mg-Al HTLs and Ca–Mg-Al HTLs mixed oxides, with a small fraction of CaCO_3_. These components demonstrated a high rate of reaction upon exposure to both HCl and SO_2_ (Reactions 4~10). As the temperature increased to 550 °C, an increased concentration of CaCO_3_ was observed, resulting from the HTLs’ decomposition. Calcium carbonate, however, exhibits comparatively lower reactivity with sulfur dioxide at elevated temperatures, thereby resulting in a marked reduction in SO_2_ adsorption efficiency. The introduction of HCl–SO_2_ mixed gases into the reactor enhanced SO_2_ removal efficiency, attributed to the formation of CaCl_2_ (Swpu 2017). This compound effectively extends the duration of Reactions 12 to 14:

12 $${\text{Ca-Mg-Al HTLs+SO}}_{2}\to {\text{Ca-SO}}_{4}{\text{ HTLs+CO}}_{2}{+ \text{H} }_{2}{\text{O}}$$

13 $${\text{Ca-Mg-Al mixed oxides+SO}}_{2}\to {\text{Ca-Mg-Al mixed sulfate+H}}_{2}{\text{O}}$$

14 $${\text{CaO+2SO}}_{2}\to {\text{CaSO}}_{3}$$

## Conclusions

In order to comprehensively explore the impact of various configuration methods on sorbent properties, an investigation into the adsorption capacities of HTLs was conducted. HTLs were synthesized under different conditions, varying calcium contents, pH values, and crystallization temperatures, followed by an evaluation of their adsorption properties using a fixed-bed setup. The findings underscored the pivotal role of sorbent structure in shaping adsorption properties. It was observed that Ca–Mg–Al mixed oxides with superior structural characteristics and crystalline forms, obtained under specific optimal conditions, exhibited significantly improved dechlorination performance, with capacity of 663.49 mg/g and adsorption rate of 0.61 mg/(g min). The introduction of calcium into the sorbent composition notably enhanced its activity by increasing diffusion, ion exchange, and cation exchange.

Furthermore, the thermogravimetric experimental outcomes indicated that the reaction temperature had a limited influence on the removal of HCl. Interestingly, as the temperature increased, the removal efficiency for HCl also improved. Conversely, the removal efficiency of SO_2_ decreased with rising temperature. Specifically, at temperatures of 350 °C and 450 °C, SO_2_ had no significant influence on the removal of HCl, allowing the Ca–Mg–Al HTLs to efficiently remove both HCl and SO_2_ concurrently with high removal efficiency. Additionally, it was observed that HCl gas promoted the SO_2_ adsorption efficiency when the temperature reached 550 °C, largely attributable to the generation of CaCl_2_.

Our investigation reveals unprecedented efficiency in the simultaneous removal of HCl and SO_2_, setting a new benchmark for sorbent performance in gas purification processes. Additionally, identifying the optimal synthesis conditions for Ca–Mg–Al mixed oxides leads to the creation of more efficient and customized sorbent materials, representing a substantial breakthrough in environmental remediation technologies.

## Data Availability

The datasets used and/or analyzed during the current study are available from the corresponding author on reasonable request.
